# Emerging Trends for Radio-Immunotherapy in Rectal Cancer

**DOI:** 10.3390/cancers13061374

**Published:** 2021-03-18

**Authors:** Claudia Corrò, Valérie Dutoit, Thibaud Koessler

**Affiliations:** 1 Translational Research Center in Onco-Hematology, Department of Medicine, Faculty of Medicine, University of Geneva, 1205 Geneva, Switzerland; Claudia.Corro@unige.ch (C.C.); Valerie.Dutoit@unige.ch (V.D.); 2 Swiss Cancer Center Léman, 1005 Lausanne, Switzerland; 3 Department of Oncology, Geneva University Hospital, 1205 Geneva, Switzerland

**Keywords:** rectal cancer, radiotherapy, immune checkpoint inhibitors, tumor microenvironment

## Abstract

**Simple Summary:**

Despite recent advances in understanding the molecular biology of tumors, obstacles still exist in the treatment of patients affected by rectal cancer. Recent evidence shows that ionizing radiation may have profound immunostimulatory effects, hinting at the possibility of exploiting radiotherapy to boost anti-tumor immunity. A bulk of work in pre-clinical tumor models have highlighted the potential benefit of this approach. Following these results, a few clinical trials are now evaluating the combination of radiotherapy and immune checkpoint inhibition. Remarkably, encouraging safety and toxicity profiles from these studies indicate that radio-immunotherapy combinations could represent a valid opportunity for rectal cancer patients. Yet, the biological and clinical impact of a radio-immunotherapy combination in rectal cancer remains unclear and further studies need to be performed to optimize the effect of these combinations.

**Abstract:**

Rectal cancer is a heterogeneous disease at the genetic and molecular levels, both aspects having major repercussions on the tumor immune contexture. Whilst microsatellite status and tumor mutational load have been associated with response to immunotherapy, presence of tumor-infiltrating lymphocytes is one of the most powerful prognostic and predictive biomarkers. Yet, the majority of rectal cancers are characterized by microsatellite stability, low tumor mutational burden and poor T cell infiltration. Consequently, these tumors do not respond to immunotherapy and treatment largely relies on radiotherapy alone or in combination with chemotherapy followed by radical surgery. Importantly, pre-clinical and clinical studies suggest that radiotherapy can induce a complete reprograming of the tumor microenvironment, potentially sensitizing it for immune checkpoint inhibition. Nonetheless, growing evidence suggest that this synergistic effect strongly depends on radiotherapy dosing, fractionation and timing. Despite ongoing work, information about the radiotherapy regimen required to yield optimal clinical outcome when combined to checkpoint blockade remains largely unavailable. In this review, we describe the molecular and immune heterogeneity of rectal cancer and outline its prognostic value. In addition, we discuss the effect of radiotherapy on the tumor microenvironment, focusing on the mechanisms and benefits of its combination with immune checkpoint inhibitors.

## 1. Introduction

### 1.1. Epidemiology of Rectal Cancer

Colorectal cancer (CRC) is the fourth most common cancer worldwide, contributing to over two million new cases and 1 million cancer-related deaths in 2018 [1,2]. Of these, one-third is represented by rectal cancer (RC) [3]. Incidence rates of CRC have been steadily rising, particularly in developing countries, probably due to the westernization of lifestyle conditions and consequent exposure to risk factors (e.g., obesity, alcohol and limited physical activity) [4]. Additionally, non-modifiable predisposing factors include hereditary genetic alterations, sex and age. CRC indeed occurs more frequently in men than women and its incidence positively correlates with age [4]. In addition, germ-line mutations in the *APC*, *BRCA*, *PTEN* and *MLH* genes are responsible for inherited syndromes such as hereditary non-polyposis CRC and several polyposis conditions [5,6].

Despite the fact that the term CRC has long been used to identify tumors of the large intestine, growing evidence suggests that colon cancer (CC) and RC are distinct tumor entities [7]. Differences exist in terms of embryonic origin, anatomic location, microbiota, macroscopic and histopathological appearance and patient management [8]. While CC encompasses the large bowel, RC is restricted to the 15 cm of the distal colon between the sigmoid and the anal margin [9]. An accurate determination of the distance to the anal verge is crucial for patient stratification and the European Society for Medical Oncology (ESMO) classifies RC into upper (10–15cm), medium (5–10cm) and lower (<5 cm) third [10]. On the other hand, the Union for International Cancer Control (UICC) sets different cutoff values by 12–16 cm as high, 6–12 cm as mid and <6 cm as low RC [11]. Although CC incidence is greater than RC, in relation to the length, the rectum mucosa has a four times higher carcinogenic risk than the colon mucosa [8]. Consequently, different growing patterns have been observed, with the appearance of polypoid non-depressed lesions more frequently seen in CC than RC [12]. Based on venous drainage, metastatic dissemination in RC involves lungs and bones more frequently than for CC, whereas CC favors the liver [13]. Ultimately, treatment modalities differentially characterize localized CC and RC: the most common option for CC is upfront surgery followed by adjuvant chemotherapy, while neoadjuvant radiotherapy (RT) or chemo-radiotherapy (CRT) followed by surgery and, eventually, adjuvant chemotherapy is used in RC [14]. Whereas extensive literature is available for CRC, this review aims to spotlight RC biology and its treatment.

### 1.2. Molecular Pathogenesis of Rectal Tumors

**Figure 1:**
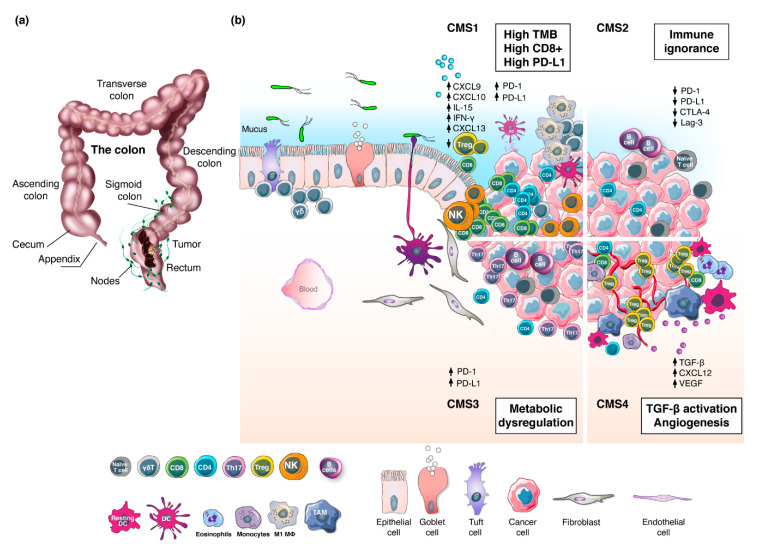
The rectal tumor microenvironment. (**a**) Illustration representing the anatomy of the large intestine and the development of a tumor localized in the rectum. (**b**) The four consensus molecular subtypes and their specific stroma-immune microenvironments. CMS1 cluster displays strong immune activation with high levels of CD8^+^ T cells, CD4^+^ T cells, γδ T cells, activated dendritic cells (DCs), natural killer (NK) cells and M1 macrophages alongside high expression of cytokines, PD1, PD-L1 and MHC-I. CMS2 shows an immune-desert microenvironment characterized by a few immune cells and poor expression of PD1, PD-L1, LAG-3 and CTLA-4. CMS3 is distinguished by metabolic dysregulation, infiltration of Th17 cells, naive B and T cells, and expression of MHC-I, PD1 and PD-L1. CMS4 exhibits high angiogenesis activity, expression of TGF-β, and infiltration of CD8^+^ T cells, CD4^+^ T cells, Tregs, M2 macrophages, monocytes, eosinophils and resting DCs.

RC arises from the malignant transformation of the epithelial cells lining the large intestine (Figure 1a) [15]. The first step of RC carcinogenesis involves the acquisition of genetic and epigenetic changes that promote proliferation [16]. These hyper-proliferative cells give rise to a benign adenoma, which may then evolve into carcinoma and spread to distant organs [17]. The latter occurs in approximately 20–30% of the cases [18], and 5-year survival for these patients is 20–40% [19]. In 1990, Vogelstein and colleagues elucidated the step-wise accumulation of genetic driver events leading the transition from adenoma to carcinoma [17]. This model describes the sequential inactivation of tumor suppressor genes (e.g., *APC*, *SMAD4*, and *P53*) and activation of oncogenic *KRAS* [20]. Thereafter, several genomic and epigenomic studies have contributed to a deeper understanding of the molecular pathogenesis of CRC at the gene-expression level and patients have been classified into two groups: microsatellite instability-high (MSI-H) and microsatellite stable (MSS). The MSI-H group accounts for 15% of the cases and is characterized by defects in the DNA mismatch repair program (dMMR), resulting frequently in high tumor mutational burden (TMB). Within this group, MSI-H sporadic CRC (67%) is caused by epigenetic inactivation of the *MLH1* gene whereas the remaining 33% is caused by germline mutations in MMR genes (particularly *MLH1* and *MSH2*) leading to hereditary non-polyposis CRC (HNPCC) [21]. The MSS group accounts for the remaining 85% of all CRCs and exhibits chromosomal instability (CIN), proficient DNA mismatch repair mechanisms (pMMR) and low TMB [22,23,24,25]. Notably, a gradient in dMMR/MSI-H has been observed in the right colon (22.3%) compared to left (4.6%) and the rectum (0.7%) [26]. Subtype analysis of the TCGA datasets revealed that, within the microsatellite classification, CC and RC have different immune microenvironments [27]. Integration of immune and transcriptomic profiling by the CRC Subtyping Consortium (CRCSC) established four consensus molecular subgroups (CMS) [28]. To date, CMS represent the best description of CRC heterogeneity (Figure 1b) [29], albeit other classifications have also been proposed [30,31]. The highly immunogenic CMS1 subtype (14%) is characterized by high TMB, dMMR/MSI-H and strong immune activation, the canonical CMS2 group (37%) displays WNT and MYC signaling activation and immune ignorance, the metabolic CMS3 class (13%) is distinguished for metabolic dysregulation, whereas the mesenchymal CMS4 subtype (23%) shows prominent transforming growth factor-β (TGFβ) activation, stromal invasion and angiogenesis. Interestingly, CMS profiles showed a decrease in CMS1 and CMS3 and an increase in CMS2 prevalence moving distally towards the rectum, while CMS4 remained relatively stable [32]. Despite recent advances in understanding the biological and molecular characteristics underpinning RC heterogeneity, the clinical significance of the CMS classification is still under evaluation [33].

## 2. Therapeutic Management of Rectal Cancer

### 2.1. Conventional Treatment Options

Surgical resection is the curative modality for the treatment of RC [34]. On top of surgery, management of RC patients largely relies on conventional methods such as chemotherapy and RT [35]. Local relapse represents a major surgical and therapeutic challenge for RC patients [36]. Because of the topographic position, innervation and vascular supply, the only curative possibility lies in an extensive surgery—abdominoperineal resection—removing the anus, the rectum, and part of the sigmoid colon along with the associated regional lymph nodes. The end of the remaining large intestine is brought out permanently as an opening, called colostomy, on the surface of the abdomen. This life-saving procedure is demanding for the surgeon and has a massive impact on the quality of life of RC patients.

Neo-adjuvant RT has been shown to reduce tumor burden, improve operative procedures and prevent local tumor recurrence [37,38,39,40]. For instance, short-course preoperative RT (SCPRT) showed a significant reduction in local recurrence compared to postoperative RT [40,41]. Similarly, preoperative CRT exhibited greater effectiveness and reduced morbidity than postoperative RT [42]. Remarkably, a lower recurrence rate was observed in patients who underwent surgery at least four weeks after RT compared to a shorter interval (≤1 week), providing the tumor adequate time to regress, allowing for tumor adaptive immune responses and enabling patients to recover from acute radiation toxicities [43,44]. Altogether, these studies contributed to the acceptance of neo-adjuvant RT and delayed surgery in clinical settings. Currently, European and American guidelines for the treatment of RC recommend five weeks neo-adjuvant CRT (45–50.4 Gy in 1.8–2 Gy/fraction, in association with fluoropyrimidine 5-fluorouracil (5-FU) radio-sensitization) followed by an 8- to 12-week treatment-free interval before radical surgery [45]. In addition to 5-FU, commonly used chemotherapies in the treatment of RC include oxaliplatin. Although they may have different mechanisms of action, these compounds share their anti-cancer effects through the disruption of DNA replication and transcription [46,47]. Alternatively, SCPRT of 25 Gy (5 × 5 Gy) with surgery taking place between 1 and 8 weeks after RT is being used [48].

Notably, RT can be administered to patients either as long-course treatment using conventional fractionation (1.8–2 Gy/fraction) or as short-course treatment using hypo-fractionation (>2 Gy/fraction) [49]. Indeed, the STOCKHOLM III study comparing long-course RT (25 × 2 Gy) with delayed surgery to short-course RT (5 × 5 Gy) and delayed surgery showed similar oncological results in terms of local recurrence, distant metastases, survival, and complications, indicating that these treatments are very similar [50]. Despite the concerns about late toxicity of hypo-fractionation, improved local tumor control and overall survival (OS) rates with minimal side effects led to the establishment of extreme hypo-fractionation regimens such as stereotactic body radiation therapy (SBRT). SBRT delivers high RT doses (≥8 Gy/fraction) in a few fractions [51], however it is not frequently used in the clinic. The biological bases for the selection of the fractionation regimen depends on the ability of the normal tissue to repair sublethal damage, tissue re-oxygenation, patients’ health conditions and concomitant therapies [52]. All these factors together determine the true biological effective dose (BED) delivered by a particular combination of dose per fraction and total dose to a particular tissue characterized by a specific α/β ratio, which reflects the sensitivity of the tissue to irradiation [53]. Consequently, the total RT dose cannot be directly compared between different fractionation regimens.

Two phase III clinical trials (POLISH and TROG 01.04) have reported a direct comparison between neo-adjuvant RT and CRT, suggesting that the two approaches are broadly comparable in their ability to lower the risk of local recurrence and so both are acceptable options [54,55]. In these trials, five-year recurrence rates for neo-adjuvant RT and CRT were 27% and 30% respectively, whereas OS rates were 74% and 70% [55]. In practice, the majority of localized RC patients are treated with SCPRT over CRT due to comparable outcomes, reduced toxicity and cost-effectiveness [56], while CRT or SCPRT with chemotherapy is favored for bigger tumors [9].

Pathological complete response (pCR) is defined as the absence of residual disease in the rectum and lymph nodes at the time of surgery [57]. In patients achieving pCR after neo-adjuvant treatment, a non-operative “watch and wait” management could be offered, hereby preserving the organ, its function and the patient’s quality of life. Up to 16% of patients receiving CRT reach pCR compared to 12% of those treated with SCPRT [48]. Patients undergoing chemotherapy before CRT or SCPRT showed increased pCR to 30–35% [58]. Similar results were observed when chemotherapy was applied after conventional neo-adjuvant RT and CRT treatments [59]. In some instances, patients achieving pCR showed lower risk of local or distant recurrence and improved survival compared to patients not achieving pCR [60], but in general there is no clear correlation between pCR and outcome. In fact, two international multicenter phase III trials (RAPIDO and PRODIGE 23) showed no survival benefit in any experimental arms where chemotherapy was applied either before or after neo-adjuvant RT or CRT compared to the conventional treatments [59,61]. Therefore, more efforts into exploring multimodality approaches should be invested.

With increasing insight into the molecular pathways governing tumor growth, the development of targeted therapies inhibiting these signals have gained attention, especially in the context of metastatic RC [62]. Despite the majority of RCs (>50%) being wild type for the *RAS* genes, targeted therapies against epithermal growth factor receptor (EGFR), which gene is mutated in a mutually exclusive manner with *RAS,* showed suboptimal activity in locally advanced RC patients [63]. On the other hand, in metastatic CRC settings, the combination of EGFR inhibitors with conventional chemotherapy showed improved progression-free survival (PFS) and OS [64,65,66]. Notably, 5% of CRCs carry a *BRAF* mutation. Remarkably, when combined with the RAF inhibitor encorafenib, which is part of the EGFR downstream signaling pathway, response rates to EGFR inhibition compared to chemotherapy alone increased from 2% to 30% and OS was improved from 5.4 months to 9 months, respectively [67]. Similarly, recent trials targeting HER2, which is a member of the EGFR superfamily, in combination with chemotherapy showed promising preliminary activity in metastatic CRC [68]. Nevertheless, anti-HER2 and anti-BRAF therapies have yet to be tested in localized RC settings. In addition to these studies, others investigated safety and efficacy of the anti-vascular endothelial growth factor (VEGF) monoclonal antibody bevacizumab with chemotherapy and CRT in CRC [69,70,71] and RC [70,71,72] showing opposite results. In particular, anti-VEGF antibody in combination with chemotherapy improved PFS and OS in metastatic CRC [69,70,71] whereas discouraging toxicity profiles were observed in phase I [70,71,72] and phase II [72] trials in locally advanced RC where anti-VEGF was combined with CRT. Despite the success of targeted therapies in advanced settings, RT followed by surgery remains the current standard-of-care for the treatment of localized RC patients.

### 2.2. Checkpoint Inhibitor Therapy

Over the past decade, the rise of immunotherapy has revolutionized the way many cancers are treated [73]. Unlike conventional therapies, these novel anti-cancer treatment modalities focus on enhancing anti-tumor T cell functions rather than targeting tumor cells directly [74]. Cancer immunotherapies include therapeutic vaccines, chimeric antigen receptor (CAR) T cell infusions [75,76], immune modulators (e.g., cytokines) and monoclonal antibodies (e.g., immune checkpoint inhibitors (ICIs)) [77,78]. The latter, in particular, have shown substantial clinical activity in patients with advanced cancers, including melanoma, non-small-cell lung cancer and CRC [79,80,81]. Although several immune checkpoints have been discovered, monoclonal antibodies against three major targets (PD1, its ligand PD-L1, and CTLA-4) have been approved for the treatment of a wide variety of cancers [82]. Expression of PD-L1 by cancer cells leads to the inhibition of T cell proliferation and cytokine secretion while simultaneously reducing apoptosis in anti-inflammatory T regulatory cells (Tregs) [83]. Similarly, CTLA-4 promotes immune escape by increasing immune tolerance [84]. By blocking these immunosuppressive interactions, ICIs reinvigorate immune recognition of tumors. Despite these successes, only a minority of patients experience clinical benefits from immune checkpoint blockade. In metastatic CRC, ICIs showed remarkable results in dMMR/MSI-H patients [85]; however, these agents have not proved meaningful activity in pMMR/MSS CRC [86]. Indeed, two phase II trials (KEYNOTE-016 and KEYNOTE-164) evaluated safety and overall response rate (ORR) of the anti-PD1 antibody pembrolizumab in pre-treated patients with advanced dMMR/MSI-H CRC [87,88]. These studies revealed durable responses in dMMR/MSI-H CRC, whereas no effect was observed in pMMR/MSS CRC (ORR of 57% and 0%, respectively) [87,88]. Similarly, the CHECKMATE-142 trial testing the effect of another anti-PD1 agent, nivolumab, showed improved clinical outcome in dMMR/MSI-H CRCs [89]. These patients displayed disease control for 12 weeks or longer, with PFS and OS rates at 12 months of 50.4% and 73.4% respectively [89]. The KEYNOTE-177 study comparing the standard-of-care first-line chemotherapy treatment to pembrolizumab in dMMR/MSI-H patients showed superiority of pembrolizumab in term of PFS (16.5 months vs. 8.2 months for chemotherapy), response rate (44% vs. 33% for chemotherapy) and quality of life [85]. Following these data, the Food and Drug Administration (FDA) granted accelerated approval for the use of pembrolizumab and nivolumab in first- and second-line treatment, respectively, of unresectable or metastatic dMMR/MSI-H CRCs.

Further studies explored the combination of immunotherapies in dMMR/MSI-H metastatic CRC. Combining the anti-CTLA-4 agent ipilimumab and nivolumab led to 80% disease control rate (stable disease) at 12 weeks and PFS and OS rates at 12 months of 71% and 87% respectively [90], outperforming results published with first-line chemotherapy or immunotherapy with single agents. This combination has been approved by the FDA in second-line treatment of dMMR/MSI-H metastatic CRCs [91]. Similarly, the NICHE study tested the combination of ipilimumab and nivolumab in the neoadjuvant setting in dMMR/MSI-H or pMMR/MSS early-stage CRC [92]. Interestingly, 27% of patients with pMMR/MSS tumors displayed pathological responses ranging from partial to complete response, providing the first indication of a clinical benefit of immune checkpoint inhibition in pMMR/MSS patients [92]. In the context of RC specifically, a phase II clinical trial (iSCORE) is currently evaluating the combination of nivolumab and the anti-LAG3 antibody relatlimab in metastatic *RAS/RAF* wild type tumors (NCT03867799).

## 3. The Prognostic Value of the Tumor Microenvironment

The network of interactions established by immune cells, endothelial cells, stromal fibroblasts and matrix-associated molecules within the tumor and the surrounding tissues generates the tumor microenvironment (TME) [93]. There is substantial evidence recognizing the TME as a determining factor for disease progression, therapeutic responses and patient outcome [7,94]. While alterations to the TME composition during tumor development have been extensively described in other reviews [95,96], our manuscript will focus on discussing the prognostic and clinical value of these interactions in the light of advancing treatment options for RC patients.

### 3.1. The Immunoscore

The importance and decisive role of the immune microenvironment in determining tumor fate has been elegantly described in a recent study by Devaud et al., where the authors identified two opposing immune response profiles with either pro- or anti-tumor properties that establish at early stage of tumor development and determine tumor progression vs. rejection [97]. Phenotypic differences in T cell infiltration within the TME can be summarized into three categories: the “immune-inflamed/hot” phenotype, in which CD8^+^ T cells infiltrate the tumor; the “immune-excluded” phenotype, in which infiltrating CD8^+^ T cells accumulate in the tumor stroma and the “immune-desert/cold” phenotype, in which CD8^+^ T cells are low or absent from the tumor and the stroma [98,99]. As T cell infiltration is typically dictated by the presence of tumor-specific antigens (neoantigens), it is not surprising that dMMR/MSI-H tumors display a greater number of tumor-infiltrating lymphocytes (TILs) as compared to pMMR/MSS cancers [100]. The presence of TILs has been shown to correlate with improved outcomes in many cancers, including RC [101,102].

In line with these observations, Galon and colleagues demonstrated the prognostic value of CD3^+^ and CD8^+^ TIL densities in advanced CRC [103]. They conducted genomic analyses and in-situ immunohistochemistry, highlighting a positive correlation between the presence of markers for Th1 polarization and of cytotoxic and memory T cells (CD3, CD8, GZMB, and CD45RO) and a low incidence of tumor recurrence. Similarly, Tosolini et al. revealed that Th1-associated gene expression is linked to a positive clinical impact in CRC patients [104]. Following this work, an international validation study demonstrated that this “immunoscore” is an independent positive prognostic biomarker for recurrence in stage I-III CRC, outcompeting the MSI status [105,106]. Interestingly, CD8^+^ and CD3^+^ T cells but not FoxP3^+^ Tregs were found to correlate with frameshift mutations in dMMR/MSI tumors [107,108]. High CD8^+^ levels and CD8^+^/FoxP3^+^ TIL ratio inversely correlated with pathological stages [109,110], whereas a positive correlation was described between FoxP3 expression and nodal dissemination and disease burden [111]. Similarly, low CD3^+^/FoxP3^+^ TIL ratio predicted adverse outcomes [112]. Since the validation of the “immunoscore”, several teams have worked towards its refinement [30]. Interestingly, expression of PD-L1 in the tumor was found to be associated with a high “immunoscore” [113]. While the prognostic value of CD3^+^ and CD8^+^ T cells is well described, the significance of Tregs remains controversial. For instance, a study in CRC showed that a high infiltration rate of FoxP3^+^ Tregs at the tumor invasive front correlated with a significantly improved prognosis [114]. Later, Lin et al. identified two Tregs populations that were associated with distinct clinical outcomes, which could explain these contrasting results [115]. These findings were corroborated by Saito and colleagues, revealing that FoxP3^high^ CD45RO^−^ but not FoxP3^low^ CD45RO^−^ Tregs were associated with poor prognosis [116]. In addition, a detailed investigation of CD3^+^ T cells in CRC liver metastasis revealed the existence of distinct spatial distribution patterns of immune cells in relation to the tumor margin that were associated with specific prognostic outcomes [117]. For instance, a significant decreased in GZMB CD8^+^ T cell density at 20–30 μm from the tumor epithelium concomitant with an increase in CD163 macrophages correlated with improved OS [117]. On the contrary, high T cells and low CD163 macrophages, which were observed at the direct tumor border <10 μm, also correlated with improved OS [117]. Taken together, this study highlights the impact of T cell function and localization on clinical responses. In the light of the recent findings, further improvements are to be expected.

### 3.2. Other Immune Cells

In addition to T cells, other immune cell populations are commonly present in the TME in RC. Natural killer (NK) cell infiltration, in particular CD56^bright^ cells, positively correlates with patient survival [118,119]. However, expression of inhibitory receptors was shown to impair NK cytotoxic function and was found to be associated with tumor progression [120]. The presence of mast cells has been associated with reduced survival and advanced pathological stages, especially in CRC liver metastasis [121,122]. Elevated neutrophil to lymphocyte ratio in the peripheral blood of advanced CRC patients has been correlated with poor clinical outcome [123], whereas the opposite has been shown for tumor eosinophil infiltration [124].

The prognostic significance of tumor-associated macrophages (TAMs) in RC remains controversial [125]. A bulk of studies indicate a positive association with survival [126,127,128], whereas others predict poor patient outcome [129,130]. Nearchou and colleagues applied automated image analysis and machine learning approaches to evaluate the prognostic significance of immune cell subpopulations and their spatial interactions in RC and found that a low CD68^+^/CD163^+^ cell ratio was significantly associated with improved DFS [131]. In contrast, Feng et al. and Yang et al. showed that high CD163^+^/CD68^+^ and CD206^+^/CD68^+^ ratios correlated with decreased relapse-free survival and OS in CRC [132,133]. Similarly, dendritic cells (DCs) exhibited positive as well as negative contributions to patients’ survival, indicative of the intrinsic heterogeneity and plasticity of these populations [134,135,136]. Finally, a high frequency of myeloid-derived suppressor cells (MDSCs) correlated with advanced stage and metastasis in the lymph nodes [137]. Interestingly, MDSCs isolated from the peripheral blood of CRC patients were able to inhibit T cell expansion in vitro [138]. Taken together, these studies show that a pro-inflammatory TME characterized by CD8^+^ T cell infiltration is associated with improved clinical outcomes in RC. In contrast, the immunosuppressive functions of other immune cells, such as MDSCs, immunosuppressive/M2 TAMs and Tregs, appear to play a major role in promoting tumor immune escape [139].

## 4. Biomarkers of Response to Immunotherapy

The positive correlation between the presence of TILs in the TME and good prognosis in RC further supports the idea that a treatment based on immunotherapy should provide clinical benefit [140]. Indeed, failure in achieving durable responses to immune checkpoint inhibition is often associated with scarce or absent T cell infiltration, indicative of an “immune-desert/cold” tumor microenvironment. The relationship between mutational load, microsatellite status and response to immunotherapy has been described in many solid cancers [141]. Along with the evidence that dMMR/MSI-H tumors are more responsive to ICIs, a significant association was predicted within the four CMS subtypes in CRC [142,143]. The highly immunogenic CMS1, characterized by elevated TMB, MSI and immune activation, correlated with good prognosis and was predicted to respond to checkpoint blockade. On the other hand, the immune ignorant CMS2 was shown to be associated with worse prognosis and was predicted to respond poorly to immunotherapy [142,143]. Although the microsatellite status is important, it is however not sufficient to guide immunotherapy responses. For instance, even within pMMR/MSS CRC, tumors with a high neoantigen load display increased T cell infiltration, which positively correlates with survival, suggesting that not all pMMR/MSS tumors are non-immunogenic [144]. Interestingly, the majority of these pMRR/MSS tumors show POLE mutations, which are known to lead to high TMB [145]. In this line, the pMMR/MSS RC patients who responded to the combination of PD1 and CTLA-4 checkpoint inhibitors showed high TMB, neoantigen load and TIL infiltration [92]. Furthermore, the authors found that CD8^+^ and PD1^+^ T cells were predictive of response [92]. Similarly, Llosa and colleagues reported that pMMR/MSS tumors responding to pembrolizumab showed an immune microenvironment resembling that of the dMMR/MSI-H tumors and were characterized by PD1^+^ CD8^+^ T cell infiltration and high levels of PD-L1 [146]. Nevertheless, in CRC, PD1/PD-L1 expression is poorly correlated with clinicopathological parameters such as tumor stage and grade [147] and the identification of more precise and reliable predictive biomarkers continues to be an unmet clinical need.

It appears increasingly clear that, in order to broaden the application of promising ICIs and develop an effective treatment strategy especially in the context of “non-inflamed/cold” tumors, novel therapeutic approaches aiming at reconditioning the TME by either promoting TIL activation and infiltration or by inhibiting immunosuppressive signals are required. To this end, cytotoxic agents such as chemotherapy and RT might affect the interplay between cancer cells and immune cells in the TME.

## 5. The Effect of Conventional Therapies on the Tumor Microenvironment

**Figure 2:**
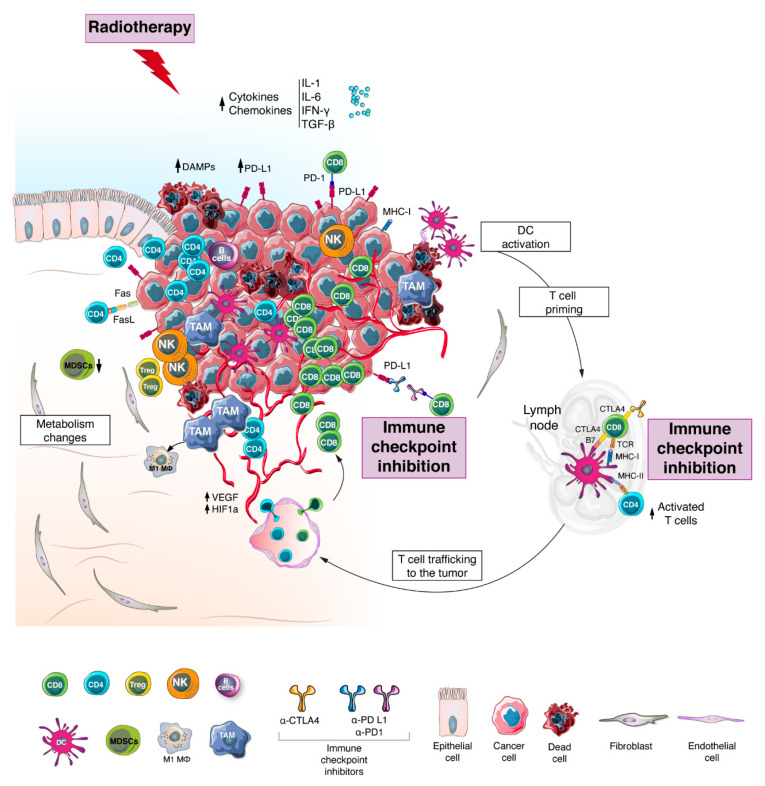
Tumor microenvironment after radiotherapy and immune checkpoint Inhibition. Upon radiotherapy, cancer cells undergo immunogenic cell death that is associated with the release of damage associated molecular patterns (DAMPs), neoantigens and pro-inflammatory cytokines (e.g., IFN-γ, IL-1 and IL-6), which promote the expression of immunomodulatory genes including antigen presentation genes and lead to the recruitment and activation of dendritic cells (DCs). Following migration to the lymph node, DCs are involved in priming and activation of T lymphocytes, which are then recruited into the tumor site alongside other immune cells. Additional modifications in the tumor microenvironment (TME) upon radiotherapy (RT) treatment include shift in macrophage phenotype towards M1, modulation of the tumor vasculature and alteration of the cell metabolism. Altogether these events enhance the recognition and killing of tumor cells. Besides inducing de novo inflammation, RT is also responsible for increasing the expression of immune checkpoints such as PD1, PD-L1 and CTLA-4. In this context, the application of immune checkpoint inhibitors might further add to the ongoing adaptive anti-tumor immunity. Taken together, the effect of RT on the TME could evoke the transition from CMS2-like TME to CMS1-like TME.

A bulk of pre-clinical work showed that modification of the TME can be achieved by chemotherapy, RT and/or targeted agents [148]. Recent evidence suggests that ionizing radiation can induce important immunomodulatory effects that may stimulate an in-situ vaccine phenomenon by reconditioning the TME and inducing T cell trafficking to the tumor [149]. Upon irradiation, cancer cells undergo immunogenic cell death that is associated with the release of damage associated molecular patterns (DAMPs), accumulation of cytosolic DNA, and upregulation of signals (e.g., IFN-γ, IL-1 and IL-6) that promote the recruitment of DCs, and expression of immunomodulatory genes including antigen presentation genes (Figure 2) [150,151].

### 5.1. Effect of Radiotherapy

In syngeneic mouse models of CRC, high dose irradiation (1 × 10 Gy and 1 × 30 Gy) promoted the expansion of effector CD8^+^ T cells and a loss of MDSCs via activation of antigen cross-presenting DCs, secretion of IFN-γ, and activation of CD4^+^ T cells expressing CD40L [152,153]. Altogether, these alterations resulted in improved tumor control in vivo [152,153]. On the other hand, high dose RT (1 × 10 Gy) was shown to increase the immunosuppressive function of Tregs in the TME via TGFβ and IL-33 signaling [154]. In RC patients, a decrease in the CD8^+^/FoxP3^+^ TIL ratio was observed after conventional as well as hypo-fractionated RT and positively correlated with OS [155]. These results are difficult to interpret and the prognostic and predictive significance of Tregs in RC remains controversial [115]. Furthermore, it appears increasingly clear that RT holds stimulatory as well as immunosuppressive traits [156], and that the fine balance between these properties dictate patient outcome. The effect of RT in promoting a pro- or an anti-inflammatory phenotype likely depends on dose, fractionation, and tumor type [157,158,159]. It has been hypothesized that high dose radiation is involved in the direct killing of primary tumor cells, which allows neoantigen release and T cell priming, whereas low dose radiation modulates the TME including at distant sites (abscopal effect) and enhances TIL infiltration, which leads to enhanced immune-cell recognition, tumor cell killing and antigen release [160]. Indeed, Dewan et al. showed that a single fraction of 20 Gy failed to inhibit tumor growth at a distant site compared to fractionated RT (3 × 8 Gy, 5 × 6 Gy) [161]. Remarkably, compared to conventional fractionated RT, hypo-fractionated RT further diminished the ratio of CD8^+^/FoxP3^+^ TILs in RC patients, indicating that differences can be observed even within fractionated regimens [155]. Grapin and colleagues showed that different fractionation schemes with similar BED (18 × 2 Gy, 3 × 8 Gy and 1 × 16.4 Gy) induced different lymphoid and myeloid responses as well as modulation of PD-L1 and TIGIT expression [162]. In particular, mice bearing subcutaneous CT26 colon tumors displayed the highest number of GZMB CD8^+^ T cells and M1/M2 TAM ratio concomitantly with the lowest CD8^+^/Treg ratio when irradiated with 3 × 8G y [162]. Contrary to the hypo-fractionated schemes, the conventional fractionated regimen (18 × 2 Gy) appeared to be the least effective [162]. Similarly, PD-L1 expression was found to be upregulated in the TME after high dose RT (1 × 12 Gy) in vitro and in vivo [163], while another pre-clinical study in syngeneic mouse model of CC demonstrated that 5 × 2 Gy RT leads to tumor cell expression of PD-L1 via IFN-γ production by infiltrating CD8^+^ T cells [164].

SCPRT (2 × 5 Gy) was also found to be associated with a shift in the polarization of TAMs towards an M1-like pro-inflammatory phenotype in vitro and in vivo [165]. Similarly, in another study, irradiated macrophages (5 × 2 Gy) showed a reduced anti-inflammatory profile, increased phagocytosis and unaltered pro-invasive and pro-angiogenic capacities [166]. Taken together, the studies presented above hints towards the possibility that hypo-fractionated RT might have a superior therapeutic potential compared to conventional fractionated RT. Nevertheless, these reports are not directly comparable as they investigated different fractionation regimens, which prevent us from drawing definitive conclusions. Therefore, the optimal radiation regimen (total dose and fractionation schedule) to stimulate an efficient anti-tumor immune response in RC remains unclear and additional work should focus on dissecting the exact kinetics and nature of how RT changes the TME.

### 5.2. Effect of Chemotherapy and CRT

The prognostic value of CD4^+^, CD8^+^ and FoxP3^+^ TILs in response to neoadjuvant chemotherapy and CRT has been extensively described [167,168,169]. Reduction in FoxP3^+^ Tregs and increased expression of MHC-I genes were observed in RC patients responding favorably to FOLFOX chemotherapy [170]. Similarly, in another study, FoxP3^+^ TILs correlated with poor therapeutic responses to FOLFOX chemotherapy alone or in combination with RT (25–50 Gy) [171]. Remarkably, in this study, neo-adjuvant CRT showed superior OS compared to chemotherapy alone, suggesting the importance of ionizing radiation in remodeling the TME [171]. In addition, a slight but not significant increase in the expression of CTLA-4 was observed upon CRT compared to chemotherapy, concomitantly to the expansion of FoxP3 Tregs [171]. These results are in line with previous reports where RT was shown to increase the proportion of Tregs, which may, in turn, induce the expression of CTLA-4 through various pathways [159,172]. On the other hand, a significant increase of CD3^+^ and CD8^+^ immune infiltrates in the TME of RC patients after neoadjuvant CRT was prognostic for the extent of tumor regression, distant metastasis rates and DFS [173]. In contrast, a multivariate analysis revealed that, while the total number of CD3^+^ and CD8^+^ TILs was significantly lower in RC after neo-adjuvant CRT compared to primarily resected cases, the level of GZMB^+^ CD8^+^ T cells was increased and positively correlated with tumor regression and lower recurrence [174]. These data suggest that the presence of GZMB^+^ CD8^+^ T cells in the TME may limit the occurrence of tumors and improve prognosis in RC patients. Alongside high levels of CD8^+^ TILs, the expression of immune checkpoint genes such as *B7-H3* and *B7-H5* was found upregulated upon CRT in RC patients and correlated with better outcome [175]. Similarly, high expression of PD-L1 in tumor nests was associated with favorable outcomes after neoadjuvant chemotherapy and CRT [171,176]. In addition, patients with high cyto-HMGB1 translocation and/or PD1^+^ TILs before treatment showed better disease control upon neo-adjuvant CRT [177]. Recent studies implied that high tumor PD-L1 expression is involved with the feedback mechanism caused by the induction of IFN-γ due to RT intervention [178,179]. Among other functions, IFN-γ has been shown to play an important role in the recruitment of T cells and expression of MHC-I through autocrine and paracrine signaling [180]. Taken together, RT has been shown to induce a two sided effect by recruiting immune cells into the tumor site and upregulating immune checkpoints. These results hint towards a potential therapeutic synergism between irradiation and immunotherapy.

## 6. Combining Radiotherapy with Immune Checkpoint Inhibitors

Combining chemotherapy with RT was shown to improve pCR in RC patients [181]. In addition, neo-adjuvant CRT has been shown to upregulate PD-L1 and promote T cell infiltration [182,183,184]. However, the combination of chemotherapy with RT often results in increased toxicity in normal tissues [185]. On the other hand, the potential of combining immunotherapy and RT has been increasingly investigated in many solid cancers, highlighting the benefit of such a combination [186,187,188]. The biological premise behind this strategy is that the release of immune-stimulating signals and neoantigens following RT will induce profound changes in the TME and promote anti-tumor immune responses that could be enhanced further by systemic immune-stimulating agents such as ICIs [189]. An overview of these alterations is outlined in Figure 2.

Deng et al. found that the combination of high dose RT (1 × 12 Gy) and anti–PD-L1 treatment induced tumor regression in a murine xenograft model of CRC [163]. In this work, radio-immunotherapy stimulated CD8^+^ T cell responses and reduced the local accumulation of MDSCs through TNF-α secretion [163]. This first study provided pre-clinical evidence of the promising effects of such a combination. Importantly, the effect of RT fractionation has been evaluated in the context of radio-immunotherapy in several studies. In a syngeneic mouse model of CC, Grapin and colleagues showed that, compared to 18 × 2 Gy and 1 × 16.4 Gy, 3 × 8 Gy irradiation induced the greatest increase in TILs and expression of PD-L1 and TIGIT and this protocol was even more effective in controlling tumor development when associated with anti-PD-L1 and anti-TIGIT treatment [162]. Similarly, combination of RT (3 × 8 Gy) with anti-CTLA-4 therapy induced tumor growth control in a MC38 xenograft model [161]. Using the same animal model, Morisada et al. showed that high dose hypo-fractionated RT (2 × 8 Gy) was superior to conventional RT (10 × 2 Gy) in enhancing anti-tumor immunity in combination with anti-PD1 treatment, highlighting the potential benefit of hypo-fractionated RT. On the other hand, two other pre-clinical studies using a CT26 xenograft model showed improved local tumor control, long-term survival, and protection against tumor re-challenge when 5 × 2 Gy RT was combined with anti-PD1 or anti-PD-L1 antibodies [164,190]. Nevertheless, the authors observed similar combinatorial activity between 5 × 2 Gy, 3 × 4 Gy and 1 × 7 Gy RT regimens even though the latter showed the poorest tumor control on its own [190]. Taken together, this study indicates that the degree to which radiation achieves local tumor control do not always predict its ability to synergize with ICIs.

Because of the differences in the dynamic progression of immunological responses upon RT and ICIs, it might be important to determine the most effective sequence of treatments [191]. Although a limited number of pre-clinical studies have been conducted on this topic, Dovedi et al. showed that anti-PD1 antibody should be administered simultaneously with RT compared to 7 days later in order to enhance its effect [164]. On the contrary, CTLA-4 inhibition was more effective when given 7 days prior to RT compared to day 1 and 5 after RT [192], probably due to the different mechanisms of action of these two immunotherapies. As CTLA-4 inhibits T cells in the early stages of the immunity cycle, it is plausible that CTLA-4 inhibition synergizes with the RT-induced enhancement of antigen-cross presentation and T cell priming by pre-conditioning the lymph node microenvironment and, subsequently, boosting T cell activation and expansion. On the other hand, PD1 acts directly on the TME; therefore, blockade of the PD1 axis promotes the activation of T cells that infiltrated the tumor site upon RT intervention [193]. In line with these observations, pre-clinical in vivo data highlighted a trajectory of immune effects such as reduction in the infiltration of MDSCs, TAMs and Tregs, and an increase in CD8^+^ T cells alongside the expression of HLA, CEA, MUC-1 and ICAM-1 reaching a peak within 8–15 days after irradiation [153,194,195]. Although optimal sequencing of RT and ICIs is not yet determined, these studies suggest that, in order to benefit from the highest levels of CD8^+^ T cells, anti-PD1 antibodies should be administered together with RT [191]. On the other hand, treatment with anti-CTLA-4 can be effective even before RT by reconditioning the lymph node microenvironment and diminishing Tregs [192].

Many clinical trials exploring the synergistic effect of combining RT with checkpoint blockade in solid cancers are ongoing [186,187,188]. In this line, a few trials are evaluating the combination of RT and immune checkpoint inhibition in RC (e.g., NCT02298946 [196], NCT02948348 [197], NCT04124601, NCT04262687, NCT04558684), but always in combination with chemotherapy. A brief summary of the relevant radio-immunotherapy clinical trials in RC is presented in Table 1. Encouraging safety and toxicity profiles from these studies indicate that radio-immunotherapy combinations could represent a valid opportunity for RC patients. For instance, in the VOLTAGE trial (NCT02948348) where patients received CRT followed by nivolumab, only mild toxicity was reported, with 7.7% of patients experiencing immune-related grade 3/4 side effects [197]. Moreover, 30% of patients with locally advanced pMMR/MSS RC reached pCR compared to 60% of dMMR/MSI-H RCs [197]. After these encouraging results, a few other trials have begun investigating the effect of such combination without the addition of chemotherapy. Remarkably, the combination of ipilimumab with SBRT (50 Gy in 4 fractions or 60 Gy in 10 fractions) has shown to induce an increase in peripheral CD8^+^ T cells and CD8^+^/CD4^+^ T cell ratio, indicating a systemic immune activation [198]. Likewise, the authors observed an increased proportion of CD8^+^ T cells expressing 4-1BB and PD1 in the tumors after treatment, which positively correlated with clinical benefit [198]. These data confirm the pre-clinical evidence on the effects of RT in remodeling the TME and are suggestive of additional immune checkpoint targets may help optimize treatment efficacy. Lastly, the combination of nivolumab and ipilimumab with RT is currently under investigation in CRC and pancreatic cancer (NCT03104439), whereas a phase II clinical trial (NCT04109755) is studying the impact of combining pembrolizumab with SCPRT in the neo-adjuvant treatment of localized pMMR/MSS RC. The results of these future clinical trials will shed light on the future of radio-immunotherapy in the treatment of RC.

## 7. Conclusions

In conclusion, the results from these first clinical and pre-clinical studies combining RT with ICIs are promising. Nevertheless, in the absence of a comprehensive understanding of the biological effect of dose/time/fractionation factors and their contribution to patient outcome, combination therapies and treatment regimens remain largely empirical. Therefore, more efforts should be directed towards designing rational and robust pre-clinical studies dissecting these aspects. A better understanding of the dynamic interaction between the immune and cancer cells and how this interaction changes after therapeutic interventions will help defining the optimal radio-immunotherapy combination to achieve the best clinical results. This may also include the addition of a third agent. For instance, Son et al. showed that injection of immature DCs in a CC xenograft model potentiated anti-tumor responses of anti-CTLA-4 therapy with RT [199]. In addition, the combination of RT with IL-12/GM-CSF and anti-PD-L1 antibody enhanced tumor regression and accumulation of CD8^+^ T cells and tumor-associated neutrophils at the primary and metastatic site in vivo [200]. Lastly, radiation combined with macrophage depletion was shown to promote adaptive immunity and potentiate checkpoint blockade [201]. While further studies need to be performed to optimize the effect of these treatment combinations, radio-immunotherapy offers an exciting new therapeutic modality for RC.

## Figures and Tables

**Table 1:** Summary of the current clinical trials in the field of radio-immunotherapy in rectal cancer.

**Short Course Radiotherapy**											
**NCT Number**	**Phase**	**Clinical Stage**	**Microsatellite Status**	**Arm**	**Neo-Adjuvant Treatment Sequence**			**Time to Surgery**	**Adjuvant Treatment**	**Nb of Patients**	**Primary Outcome**
					**Pre-Radiotherapy Treatment**	**Radiotherapy Treatment**	**Post-Radiotherapy Treatment**				
NCT04663763	II	T3–4 and/or N+	MSS	A	-	scRT (5 × 5 Gy)	4 cycles of CAPOX and anti-PD1 antibody (Sintilimab)	1 week after the end of neoadjuvant therapy	4 cycles of CAPOX	32	pCR rate
			MSI-H	B	-	scRT (5 × 5 Gy)	4 cycles of CAPOX and PD1 antibody (Sintilimab)	1 week after the end of neoadjuvant therapy	4 cycles of CAPOX	8	pCR rate
NCT04518280	II R*	T3–4 and/or N+	MSS	A	2 cycles of CAPOX and anti-PD1 antibody (Toripalimab)	scRT (5 × 5 Gy)	4 cycles of CAPOX and anti-PD1 antibody (Toripalimab)	2–4 weeks after the end of neoadjuvant therapy	-	65	pCR rate
				B	-	scRT (5 × 5 Gy)	6 cycles of CAPOX and anti-PD1 antibody (Toripalimab)	2–4 weeks after the end of neoadjuvant therapy	-	65	pCR rate
NCT04558684	II	>T2N0 or low T2N0	-	-	-	scRT (5 × 5 Gy)	6 cycles of CAPOX and anti-PD1 antibody (Camrelizumab)	8 (+/−4) weeks after the end of neoadjuvant therapy	-	30	cCR rate
NCT04621370 PRIME-RT study	II R*	cT3b+, N+, EMVI+	-	A	Anti-PD-L1 antibody (Durvalumab) week prior radiotherapy	scRT (5 × 5 Gy)	6 cycles of FOLFOX and anti-PD-L1 antibody (Durvalumab)	3–5 weeks after the end of neoadjuvant therapy	-	24	pCR rate
			-	B	Anti-PD-L1 antibody (Durvalumab) week prior radiotherapy	capecitabine radiosensitized NACRT (50Gy)	4 cycles of FOLFOX and anti-PD-L1 antibody (Durvalumab)	3–5 weeks after the end of neoadjuvant therapy	-	24	pCR rate
NCT04109755 PEMREC study	II	cT3–T4 N0 or cT any and N1-2	MSS		-	scRT (5 × 5 Gy) with anti-PD1 antibody (Pembrolizumab)	3 cycles of anti-PD1 antibody (Pembrolizumab)	3 weeks after the end of neoadjuvant therapy	-	25	TRG
NCT04231552	II	cT3–4 or N+	-		-	scRT (5 × 5 Gy)	2 cycles of CAPOX and anti-PD1 antibody (Camrelizumab)	n.a	-	30	pCR rate
NCT03503630	II	cT2 N1–3, cT3 N0–3	-		-	scRT (5 × 5 Gy)	6 cycles of FOLFOX and anti-PD-L1 antibody (COMPOUND 2055269)	2–3 weeks after the end of neoadjuvant therapy	-	44	pCR
NCT04503694 REGINA study	II	Intermediate risk MRI-defined rectal cancer	-		2 cycles anti-PD1 antibody (Nivolumab) + regorafenib	scRT (5 × 5 Gy)	3 cycles anti-PD1 antibody (Nivolumab) + regorafenib	7–8 weeks after the end of scRT therapy	-	60	pCR
NCT04636008	Ib	≥cT2	MSI-H/dMMR		-	scRT (5 × 5 Gy)	3 cycles of anti-PD1 antibody (Sintilimab)	1–2 weeks after the end of neoadjuvant therapy	-	20	TRAE rate
**Long Course Chemo-Radiotherapy**											
**NCT Number**	**Phase**	**Clinical Stage**	**Microsatellite Status**	**Arm**	**Neo-Adjuvant Treatment Sequence**			**Time to Surgery**	**Adjuvant Treatment**	**Nb of Patients**	**Primary Outcome**
					**Pre-Radiotherapy treatment**	**Radiotherapy Treatment**	**Post-Radiotherapy Treatment**				
NCT04411537	II	T3–4 and/or N+	MSS		2 cycles of anti-PD1 antibody	Capecitabine plus irinotecan radiosensitized NACRT (50 Gy)	3 cycles of anti-PD1 antibody	1–2 weeks after the end of neoadjuvant therapy	6 cycles of XELOX	50	pCR rate
NCT04411524	II	T3–4 and/or N+	MSI-H		2 cycles of anti-PD1 antibody	Capecitabine plus irinotecan radiosensitized NACRT (50 Gy)	3 cycles of anti-PD1 antibody	1–2 weeks after the end of neoadjuvant therapy	6 cycles of XELOX	50	pCR rate
NCT03854799 AVANA study	II	cN+, cT4, high risk cT3	-		-	Capecitabine radiosensitized NACRT (50.4 Gy) with anti-PD-L1 antibody (Avelumab)	-	8–10 weeks after the end of neoadjuvant therapy	-	101	pCR rate
NCT04357587	II	Stage II or stage III or olimetastatic	dMMR		-	Capecitabine radiosensitized NACRT (50 Gy) with PD1 antibody (Pembrolizumab)	1 cycle of anti-PD1 antibody (Pembrolizumab)	n.a	-	10	Rate of AE Feasability
NCT03921684	II	T3–4 N0 or TX N+	-		-	Capecitabine radiosensitized NACRT (50.4 Gy)	6 cycles of FOLFOX and anti-PD-1 antibody (Nivolumab)	4 weeks after the end of neoadjuvant therapy	-	29	pCR rate TRAE
NCT02921256	II	Stage II or stage III	-	C	8 cycles of FOLFOX	Capecitabine radiosensitized NACRT (50 Gy) with anti-PD1 antibody (Pembrolizumab)	5 cycles of anti-PD1 antibody (Pembrolizumab)	n.a	-	>100	Change in NAR score
NCT03127007 R-IMMUNE study	Ib/II R*	Stage II or stage III	-	A	-	5-FU radiosensitized NACRT (50 Gy) with anti-PD-L1 antibody (Atezolizumab)	3 cycles of anti-PD-L1 antibody (Atezolizumab)	3 weeks after the end of neoadjuvant therapy	-	54	pCR rate AE rate
			-	B	-	5-FU radiosensitized NACRT (50 Gy)	-	10 weeks after the end of neoadjuvant therapy	-		
NCT04443543	II	T2–4 and/or N+	MSS	A	-	Capecitabine plus irinotecan radiosensitized NACRT (50 Gy)	XELIRI or FOLFIRINOX adaptive number of cycles and regimen	No surgery for those in cCR	-	222	cCR rate
			MSI-H/dMMR	B	-	Capecitabine plus irinotecan radiosensitized NACRT (50 Gy)	3 cycles of anti-PD1 antibody (Tislelizumab)	No surgery for those in cCR	-	NA	cCR rate
NCT04017455 TARZAN study	II	Intermediate risk rectal cancer or low risk distal rectal cancer	-		-	Radiotherapy	3 cycles of anti-VEGF antibody (bevacizumab) combined with anti-PD-L1 antibody (Atezolizumab)	3 weeks after the end of neoadjuvant therapy	-	38	cCR rate ncCR rate
NCT04124601 CHINOREC Study	II R*	NA	-	A	-	Capecitabine radiosensitized NACRT (50 Gy)	-	n.a	-	80	TRAE rate
		NA	-	B	-	Capecitabine radiosensitized NACRT (50 Gy)	Anti-CTLA-4 antibody (Ipilimumab) on day 7 and anti-PD1 antibody (Nivolumab) on day 14, 28 and 42)	n.a	-		
NCT04293419 DUREC study	II	High risk MRI-defined rectal cancer	-		6 cycles of FOLFOX + 4 cycles anti-PD-L1 (Durvalumab)	Capecitabine radiosensitized NACRT (50.4 Gy) + anti-PD-L1 (Durvalumab)	2 cycles anti-PD-L1 (Durvalumab)	2–6 weeks after the end of neoadjuvant therapy	-	58	pCR rate
NCT03102047 FR-2 study	II	Stage II–IV rectal cancer	MSS		-	Capecitabine radiosensitized NACRT (50.4 Gy)	4 cycles anti-PD-L1 (Durvalumab)	1–4 weeks after the end of neoadjuvant therapy	-	47	NAR
NCT02948348 VOLTAGE trial	Ib/II	T3 and T4, N any	-		-	Capecitabine radiosensitized NACRT (50.4 Gy)	5 cycles anti-PD1 antibody (Nivolumab)	2 weeks after the end of neoadjuvant therapy	-	50	pCR rate
NCT03299660	II	T3bN1-N2M0, T3c/dN0-N2M0, T4N0-N2M0	-		-	Capecitabine/5FU radiosensitized NACRT (50.4 Gy)	4 cycles of anti-PD-L1 antibody (Avelumab)	8–10 weeks after the end of neoadjuvant therapy	-	45	pCR rate
NCT04083365 PANDORA study	II	cT3/4N0/M0 or Tx N1-2/M0	-		-	Capecitabine radiosensitized NACRT (50.4 Gy)	3 cycles of anti-PD-L1 antibody (Avelumab)	1–2 weeks after the end of neoadjuvant therapy	-	60	pCR rate
**Only Immune Checkpoint Inhibitors**											
**NCT Number**	**Phase**	**Clinical Stage**	**Microsatellite Status**	**Arm**	**Neo-Adjuvant Treatment Sequence**			**Time to Surgery**	**Adjuvant Treatment**	**Nb of Patients**	**Primary Outcome**
					**Pre-Radiotherapy Treatment**	**Radiotherapy Treatment**	**Post-Radiotherapy Treatment**				
NCT04643041 BASKET study	II	T × N × M0	MSI-H/dMMR		6 cycles of anti-PD1 antibody	No radiotherapy	-	No surgery	-	47	1 year DFS rate
**Other Immunotherapies**											
**NCT Number**	**Phase**	**Clinical Stage**	**Microsatellite Status**	**Arm**	**Neo-Adjuvant Treatment Sequence**			**Time to Surgery**	**Adjuvant Treatment**	**Nb of Patients**	**Primary Outcome**
					**Pre-Radiotherapy Treatment**	**Radiotherapy Treatment**	**Post-RadiotherApy treatment**				
NCT03300544	I	cT3–4, N+	-		Four injections of T-VEC with 2 cycle of FOLFOX	Capecitabine radiosensitized NACRT (50.4 Gy)	-	8–12 weeks after the end of neoadjuvant therapy		21	DLT
NCT02688712 ExIST Study	II	Stage II or stage III	-		TGFβ Type I Receptor Inhibitor (LY2157299) for 15 days	Capecitabine radiosensitized NACRT (50.4 Gy) with (LY2157299) 15 days after start	-	6–10 weeks after the end of neoadjuvant therapy		50	pCR rate
NCT04130854 INNATE study	II R*	cT4 or within 3mm of MR fascia	-	A	-	CD-40 agonist antibody with scRT (5 × 5 Gy)	6 cycles of FOLFOX and CD-40 agonist antibody (APX005M)	n.a		58	pCR rate
			-	B	-	scRT (5 × 5 Gy)	6 cycles of FOLFOX	n.a			
NCT03916510 CEDAR study	I	cT3mrf+ or N+ or low tumors	-		Three enadenotucirev loading doses in weeks 1-2	Capecitabine radiosensitized NACRT (50.4 Gy) +/− Enadenotucirev	+/− Enadenotucirev	n.a		30	DLT MRI-TRG
NCT04304209	II R*	cT3–4N0M0 or cT×N+M0	dMMR or MSI-H	A	4 cycles of anti-PD1 antibody (Sintilimab)	No Radiotherapy	4 cycles of anti-PD1 antibody (Sintilimab) +/− CAPEOX chemotherapy	Surgery or watch and wait		195	pCR
			pMMR/MSS/MSI-L	B1	4 cycles of anti-PD1 antibody (Sintilimab)	Capecitabine radiosensitized NACRT (50.4 Gy) + CAPEOX	-	Surgery or watch and wait			
				B2	-	Capecitabine radiosensitized NACRT (50.4 Gy) + CAPEOX	-	Surgery or watch and wait			

AE: adverse event; CAPOX (also called XELOX): capecitabine and oxaliplatin; cCR: clinical complete response; DLT: dose limiting toxicities; dMMR: deficient mismatch mechanisms of repair; FOLFOX: leucovorin, 5-FU and oxaliplatin; FOLFIRINOX: leucovorin, 5-FU, irinotecan and oxaliplatin; IMRT DT: 50Gy in 25 fractions; MR: mesorectal; MRF: MR fascia; MRI: magnetic resonance imaging; MSI-H: microsatellite instability high; MSS: microsatellite stable; NACRT: neoadjuvant chemo-radiotherapy; NAR: neoadjuvant rectal cancer; ncCR: near-complete response rate; pCR: pathological complete response; R*: randomized; scRT: short course radiotherapy (5 × 5 Gy); TME: total mesorectal excision; TRAE: treatment-related adverse event; TRG: tumor regression grade; XELIRI: irinotecan and capecitabine; n.a.: information not available.

## Data Availability

No new data were created or analyzed in this study. Data sharing is not applicable to this article.
